# Interaction between Mitochondria and the Endoplasmic Reticulum: Implications for the Pathogenesis of Type 2 Diabetes Mellitus

**DOI:** 10.1155/2012/242984

**Published:** 2011-07-19

**Authors:** Jaechan Leem, Eun Hee Koh

**Affiliations:** Department of Internal Medicine, University of Ulsan College of Medicine, 388-1 Poongnap-Dong, Songpa-Gu, Seoul 138-736, Republic of Korea

## Abstract

Mitochondrial dysfunction and endoplasmic reticulum (ER) stress are closely associated with **β**-cell dysfunction and peripheral insulin resistance. Thus, each of these factors contributes to the development of type 2 diabetes mellitus (DM). The accumulated evidence reveals structural and functional communications between mitochondria and the ER. It is now well established that ER stress causes apoptotic cell death by disturbing mitochondrial Ca^2+^ homeostasis. In addition, recent studies have shown that mitochondrial dysfunction causes ER stress. In this paper, we summarize the roles that mitochondrial dysfunction and ER stress play in the pathogenesis of type 2 DM. Structural and functional communications between mitochondria and the ER are also discussed. Finally, we focus on recent findings supporting the hypothesis that mitochondrial dysfunction and the subsequent induction of ER stress play important roles in the pathogenesis of type 2 DM.

## 1. Introduction

Type 2 diabetes mellitus (DM) is characterized by impaired insulin secretion from pancreatic *β*-cells. In addition, insulin-responsive tissues, such as muscle, liver, and adipose tissue, exhibit insulin resistance. A number of findings suggest that both of these major features of type 2 DM are associated with mitochondrial dysfunction and/or endoplasmic reticulum (ER) stress [1–4]. Recently, it was shown that mitochondria and the ER interact both physically and functionally [5, 6]. In this paper, we will focus on the roles that mitochondrial dysfunction and ER stress play in the pathogenesis of type 2 DM. Particular emphasis will be placed on recent findings elucidating the interaction between mitochondria and the ER. 

## 2. Role of Mitochondrial Dysfunction in Type 2 DM

### 2.1. Mitochondria

 The mitochondrion is an intracellular double-membraned organelle found in most eukaryotic cells [7]. Mitochondria are well known to be power stations within cells, as one of their major functions is production of ATP [8]. In addition, mitochondria play essential roles in intracellular reactive oxygen species (ROS) production [9], regulation of apoptosis [10], and Ca^2+^ storage [11].

### 2.2. Mitochondrial and Pancreatic *β*-Cell Dysfunction

**Figure 1:**
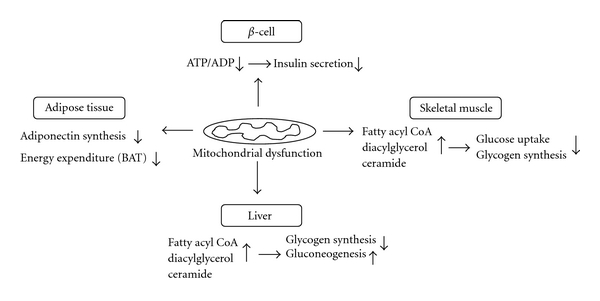
Roles of mitochondrial dysfunction in the pathogenesis of *β*-cell dysfunction and insulin resistance.

 Insulin-resistant patients can develop overt type 2 DM when pancreatic *β*-cells are unable to produce enough insulin to maintain normoglycemia. Pancreatic *β*-cells from patients with type 2 DM cannot sense glucose properly, and this contributes to impairment of insulin secretion. Interestingly, glucose sensing by *β*-cells appears to be controlled by mitochondrial metabolism. Reduced forms of nicotinamide adenine dinucleotide (NADH) or flavin adenine dinucleotide (FADH_2_) are generated during glucose metabolism via both glycolysis and the tricarboxylic acid (TCA) cycle. Electron transfer to the mitochondrial electron-transport chain (ETC) by NADH and FADH_2_ leads to production of ATP via the process of oxidative phosphorylation (OXPHOS). Increases in the ATP/ADP ratio in *β*-cells inhibit ATP-sensitive potassium channels (K_ATP_), in turn inducing depolarization of plasma membranes. The opening of voltage-sensitive Ca^2+^ channels allows Ca^2+^ uptake by *β*-cells, thereby contributing to secretion of insulin. Thus, mitochondrial dysfunction can impair glucose-stimulated insulin secretion by reducing the ATP/ADP ratio within *β*-cells (Figure 1) [12].

### 2.3. Mitochondrial Dysfunction and Skeletal Muscle Insulin Resistance

 Defective mitochondrial fatty acid metabolism in skeletal muscle is thought to affect insulin signaling pathways, thereby leading to insulin resistance [13–15]. Impairment of mitochondrial fatty acid *β*-oxidation, either alone or in conjunction with increased delivery of free fatty acids (FFAs) from plasma, leads to elevated levels of intracellular fatty acid metabolites such as fatty acyl CoA, diacylglycerol, and ceramide [16–18]. Metabolites formed under such circumstances activate serine/threonine kinases including protein kinase C (PKC), leading to phosphorylation of serine sites on insulin receptor substrate-1 (IRS-1) [19, 20]. Increased serine phosphorylation of IRS-1 inhibits the tyrosine kinase activity of the insulin receptor on IRS-1 and the activity of insulin-stimulated phosphatidylinositol 3-kinase (PI 3-kinase), resulting in decreased activity of insulin-stimulated protein kinase B (PKB, also known as AKT). Reduced AKT activity leads to suppression of insulin-stimulated glucose transporter 4 (GLUT4) translocation and subsequent reduction of glycogen synthesis (Figure 1).

### 2.4. Mitochondrial Dysfunction and Hepatic Insulin Resistance

The liver plays a crucial role in the development of insulin resistance and type 2 DM [21]. Several lines of evidence indicate that defects in liver mitochondrial oxidative function can induce hepatic insulin resistance [14, 15, 22, 23]. For example, reduced levels of mitochondrial fatty acid *β*-oxidation in the liver, as in skeletal muscle, lead to accumulation of intracellular fatty acid metabolites [24, 25]. Note that similar results were observed either when de novo hepatic lipogenesis rose or when delivery of FFAs from the plasma increased. Under either circumstance, the metabolites adversely affected intracellular insulin signaling, leading to reduced insulin stimulation of glycogen synthesis and increased hepatic gluconeogenesis (Figure 1) [19].

### 2.5. Mitochondrial Dysfunction and Adipose Tissue

Adipose tissue has been described as an endocrine organ that plays a central role in fuel metabolism [26]. Adipocytokines such as leptin, adiponectin, resistin, and tumor necrosis factor-*α* (TNF-*α*) are released by adipose tissue, and these cytokines regulate fuel metabolism [27]. Adiponectin is known to have insulin-sensitizing effects. However, in contrast to other adipocytokines, the plasma levels of adiponectin are significantly decreased in obese subjects and in type 2 DM patients [28, 29]. Recently, we reported that the levels of adiponectin in plasma and adipose tissue were significantly lowered in obese mice; an associated reduction of mitochondrial content and function in adipose tissue was also documented [30]. Rosiglitazone, a peroxisome proliferator-activated receptor *γ* (PPAR*γ*) agonist, reversed decreases in plasma adiponectin levels and adiponectin expression in obese mice, and elevated mitochondrial content and function in adipose tissue. These findings suggest that mitochondrial dysfunction in adipose tissue leads to decreased plasma adiponectin levels in obese subjects (Figure 1).

 Many studies on rodents have shown that the capacity of mitochondria for oxidizing fatty acids in brown adipose tissue (BAT) plays a critical role in the regulation of adaptive thermogenesis, energy balance, and body weight [31, 32]. The presence of BAT was considered to be relevant only in human newborn and small mammals. However, recent studies using positron-emission tomography and computed tomography (PET-CT) demonstrated that adult humans possess active BAT [33, 34]. Thus, mitochondrial dysfunction in BAT appears to be linked to impaired thermogenesis and energy expenditure, contributing to the development of obesity and insulin resistance in adult humans (Figure 1) [35].

## 3. Role of ER Stress in Type 2 DM

### 3.1. ER

 The ER is a complex organelle that is found in all eukaryotic cells. Structurally, the ER is formed by an interconnected network of cisternae and microtubules. From a functional viewpoint, the ER plays a central role in protein folding and in quality control of newly synthesized proteins [36]. The ER also serves as an essential site for synthesis of lipids [37] and for high-capacity buffering of intracellular Ca^2+^ [38].

### 3.2. ER Stress

 If proteins are to be folded properly within the ER, a balance must be struck between the ER protein load and ER folding capacity. A number of conditions can disrupt ER homeostasis, leading to accumulation of misfolded proteins within the lumen of the ER [4, 39]. Such conditions include a large biosynthetic load, defects in folding machinery, and disturbances in the handling of Ca^2+^. Accumulation of misfolded proteins in the ER causes ER stress, and this activates an elaborative adaptive process termed the unfolded protein response (UPR) [40].

The UPR is triggered by three ER transmembrane proteins: protein kinase R-like ER kinase (PERK), inositol-requiring enzyme 1 (IRE1), and activating transcription factor 6 (ATF6). In unstressed conditions, ER luminal domain of these proteins are bound by the chaperone Bip, maintaining them in an inactive state until ER stress is present [41]. During ER stress, misfolded proteins sequester, Bip, leading to free PERK and IRE1 monomers to oligomerize and trans-autophosphorylate. Activated PERK mediates inhibition of protein translation via phosphorylation of eukaryotic translation initiation factor 2*α* (eIF2*α*), resulting in reduced global protein synthesis in an attempt to decrease the protein-folding load in the ER lumen [42]. PERK-mediated eIF2*α* phosphorylation also contributes to the activation of a subset of translational targets including activating transcription factor 4 (ATF4). ATF4 activates transcriptionally the proapoptotic transcription factor CCAAT/enhancer binding protein (C/EBP) homologous protein (CHOP) [43].

Activation of IRE1, which has endoribonuclease activity, leads to splicing of X-box binding protein-1 (XBP1) mRNA and translation of the active form (XBP1s) [44]. XBP1s translocates to the nucleus and regulates expression of ER chaperones and proteins involved in ER-associated degradation (ERAD) [45]. In addition, the cytosolic domain of IRE1 can associate with TNF receptor-associated factor 2 (TRAF2) to activate the apoptosis signal-regulating kinase 1 (ASK1) and c-Jun N-terminal kinase (JNK) pathway, independently with its endoribonuclease activity [46, 47]. 

In response to ER stress, ATF6, released from Bip, translocates to the Golgi where it is cleaved by proteases into an active amino-terminal form [48]. N-terminal ATF6 in turn moves to the nucleus to stimulate expression of ER chaperones and proteins involved in ERAD.

### 3.3. ER Stress and *β*-Cell Dysfunction

ER stress plays an important role in the pathogenesis of type 2 DM, as such stress contributes to pancreatic *β*-cell dysfunction and insulin resistance [4, 49]. When the demand for insulin overwhelms the folding capacity of the ER, the UPR becomes chronically activated. Several stimuli have been shown to cause sustained accumulation of misfolded proteins within the ER lumen of *β*-cells [4]. These include high levels of FFA (caused by either a high-fat diet or obesity) and glucose (chronic hyperglycemia), as well as aggregation of islet amyloid polypeptide. Accumulation of misfolded proteins triggers chronic activation of the UPR, inducing *β*-cell dysfunction and apoptosis [50, 51].

**Figure 2:**
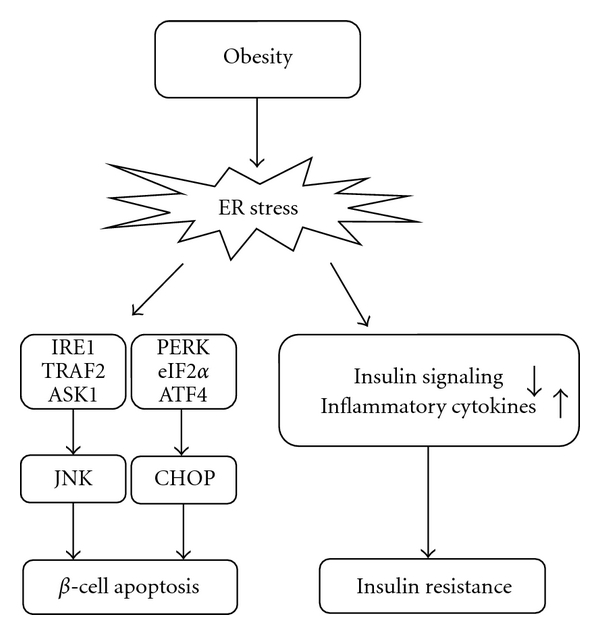
Roles of ER stress in the pathogenesis of *β*-cell apoptosis and insulin resistance.

 Several components of the UPR that contribute to *β*-cell apoptosis have been shown (Figure 2). ER stress can induce *β*-cell apoptosis through prolonged activation of IRE1-TRAF2-ASK1 cascade and JNK pathway [52]. CHOP also plays a crucial role in the induction of ER stress-mediated *β*-cell apoptosis [53].

### 3.4. ER Stress and Insulin Resistance

In addition to *β*-cell dysfunction, ER stress is involved in peripheral insulin resistance (Figure 2). Obesity results in chronic stimulation of ER stress, leading to continuous activation of the UPR. Recent studies have suggested that this may, in fact, be the main mechanism of peripheral insulin resistance and type 2 DM [3, 54]. In obese mice, the levels of ER stress markers are increased in the liver and adipose tissue [3]. Obesity-induced ER stress inhibits insulin signaling, and this leads to insulin resistance. ER stress can also activate nuclear factor-*κ*B (NF-*κ*B) signaling in the liver [55], thereby increasing production of proinflammatory cytokines and causing development of insulin resistance [56]. A recent study showed that treatment of obese diabetic mice with the chemical chaperones 4-phenyl butyric acid (PBA) and taurine-conjugated ursodeoxycholic acid (TUDCA) improved peripheral insulin sensitivity by alleviating ER stress [57]. TUDCA therapy also improved insulin sensitivity in the liver and muscle of obese subjects [58].

## 4. Structural Communication between Mitochondria and the ER

A number of studies have shown structural communication between the mitochondria and the ER. The evidence includes cosedimentation of ER particles with mitochondria, as well as electron microscopic observation of a close physical apposition between mitochondria and the ER [59, 60]. More recently, high-resolution three-dimensional images have been obtained showing an interaction between mitochondria and the ER; specific color labels were employed to this end [61]. A recent study using electron tomography also demonstrated that the outer mitochondrial membrane (OMM) and the ER are joined by tethers, enabling ER proteins to associate directly with proteins and lipids of the OMM [62].

The structural membrane hat bridges between mitochondria and the ER is known as the mitochondria-associated membrane (MAM) [63]. The MAM plays an essential role in several cellular functions, including lipid transport, Ca^2+^ signaling, and apoptosis [64]. A number of mitochondrial or ER-bound proteins are important for maintaining structural communication between the two organelles at the MAM [64, 65]. In particular, communication between the organelles is modulated by a family of chaperone proteins. The voltage-dependent anion channel (VDAC) is physically linked to the inositol 1,4,5-triphosphate receptor (IP_3_R) via the molecular chaperone grp75 [66]. Overexpression of the cytosolic form of grp75 selectively increases IP_3_-induced Ca^2+^ uptake into the mitochondrial matrix, whereas overexpression of the mitochondrial form of the protein does not have this effect. Another protein that modulates interaction between mitochondria and the ER is phosphofurin acidic cluster sorting protein 2 (PACS-2), which is known to integrate ER-mitochondrial communication and apoptosis signaling [67]. Accordingly, PACS-2 depletion induces mitochondrial fragmentation, dissociates the ER from mitochondria, and blocks apoptosis signaling. More recently, Sigma-1 receptors have been shown to be located at the MAM of the ER, where they form complexes with Bip [68]. Sigma-1 receptors dissociate from Bip and bind to type-3 IP_3_Rs under conditions of ER Ca^2+^ depletion. Thus, type-3 IP3Rs are not degraded by proteasomes. Ca^2+^ depletion appears to induce a prolonged Ca^2+^ signaling event from the ER to the mitochondria, via IP_3_Rs. Together, the data suggest that Sigma-1 receptors are involved in maintaining normal Ca^2+^ signaling from the ER to mitochondria.

Structural communication between mitochondria and the ER is also modulated by fission and fusion of mitochondria. Fission and fusion are regulated by a family of mitochondrion-shaping proteins including dynamin-related protein 1 (DRP1), mitofusin 1, and mitofusin 2 [69]. Mitofusin-2 is a mitochondrial transmembrane GTPase that regulates mitochondrial fusion [70], and this protein is enriched at MAMs [71]. Mitofusin-2 tethers the ER to mitochondria via formation of both homotypic and heterotypic complexes. For example, ER mitofusin-2 interacts with either mitofusin-2 or mitofusin-1 on mitochondria. The tethering effect of mitofusin-2 appears to play a role in the control of Ca^2+^ flow between mitochondria and the ER [71].

## 5. Functional Communication between Mitochondria and the ER

### 5.1. Role of ER Stress in Induction of Mitochondrial Dysfunction

 Mitochondrial dysfunction and ER stress have each been recognized to play crucial roles in the pathogenesis of type 2 DM. However, the individual stressors appear to act sequentially in various tissues. For example, accumulating evidence has shown that ER stress induces mitochondrial dysfunction, thereby leading to disruption of various physiological responses within cells [5, 6]. 

Interactions between mitochondria and the ER facilitate control of Ca^2+^ signaling and Ca^2+^-dependent cellular processes such as apoptosis [72, 73]. Prolonged ER stress leads to release of Ca^2+^ from the ER lumen at the MAM. In contrast, such stress leads to increased Ca^2+^ uptake into the mitochondrial matrix. Elevated Ca^2+^ uptake induces an imbalance between mitochondrial Ca^2+^ load and the buffering capacity of the matrix, and such imbalance ultimately leads to a prolonged episode of massive mitochondrial Ca^2+^ accumulation. Sustained Ca^2+^ accumulation triggers opening of the mitochondrial permeability transition pore (mtPTP). Ultimately, this results in swelling of the organelle, rupture of the OMM, and release of proapoptotic proteins into the cytosol [74].

 ROS are thought to act as local messengers between the ER and mitochondria [6]. Many ROS sources and targets are localized to the ER and mitochondria [75, 76]. Disulfide bond formation is a critical step in folding of newly synthesized proteins, and this is mediated by members of the ER oxidoreductin 1 (Ero1) family [77]. Importantly, ROS are concomitantly produced by Ero1. Previous studies have shown that Ero1 can be activated under conditions of ER stress [78, 79]. Thus, conditions that trigger such stress may lead to excessive production of ROS in the ER. Such elevated ROS levels inactivate the sarco-endoplasmic reticulum Ca^2+^ ATPase (SERCA) and activate IP_3_R via oxidation [80, 81]. Modulation of Ca^2+^ channel activity by ROS increases the level of Ca^2+^ on the cytosolic face of the ER and also promotes Ca^2+^ uptake into the mitochondrial matrix. Therefore, ROS production mediated by Ero1 provides an additional mechanism by which ER stress can induce mitochondrial dysfunction.

### 5.2. Role of Mitochondrial Dysfunction in Induction of ER Stress

#### 5.2.1. NO-Mediated Induction of the ER Stress Response via Inhibition of Mitochondrial Respiration

Protein folding processes and the handling of Ca^2+^ within the ER each require large amounts of ATP. Accordingly, ATP depletion is one of the best-known mechanisms by which ER stress may be induced [82]. Such observations have raised significant questions regarding the modes by which changes in mitochondrial function affect processes within the ER. It is widely accepted that ER stress induces mitochondrial dysfunction. However, it appears that this is not a one-way process; Xu et al. have shown that the ER stress response can be induced following disruption of the mitochondrial respiratory chain by nitric oxide [83]. 

NO can bind to cytochrome c oxidase and inhibit the enzyme, in competition with oxygen [84]. Thus, the respiratory chain is disrupted in NO-generating cells [83]. Because this process is accompanied by mitochondrial Ca^2+^ flux, disruption of electron transfer by cytochrome c oxidase may result in changes in the extent of Ca^2+^ flux between the mitochondria and the ER. NO-mediated changes in Ca^2+^ flux between these organelles increase expression of ER stress-responsive genes such as glucose-regulated protein 78 (Grp78), elevated levels of which provide significant cytoprotection against thapsigargin, a selective ER Ca^2+^ ATPase inhibitor. Interestingly, chemical disruption of mitochondrial Ca^2+^ flux has been shown to reverse NO-mediated cytoprotection. In addition, the NO-mediated ER stress response was diminished in *rho^o^* cells devoid of mitochondrial DNA [83]. Together, these results suggest that NO signals the ER stress response via inhibition of mitochondrial respiration.

#### 5.2.2. Mitochondrial Dysfunction Induces ER Stress and Decreases Adiponectin Synthesis

Recently, we showed that impairment of mitochondrial function increases the levels of ER stress markers [30]. Adenovirus-mediated overexpression of nuclear respiratory factor-1 (NRF-1), a transcription factor that regulates the expression of nuclear-encoded mitochondrial genes, reduced the upregulation of ER stress markers associated with mitochondrial dysfunction. Previous studies showed that JNK and activating transcription factor 3 (ATF3) were activated by ER stress [3, 85]. Further, impairment of mitochondrial function sequentially activated JNK and ATF3. However, inhibition of JNK and ATF3 reversed the reduction in adiponectin transcription that was induced by mitochondrial dysfunction [30]. Together, the data suggest that mitochondrial dysfunction induces ER stress. This, in turn, activates signaling cascades involving JNK and ATF3, thereby decreasing adiponectin synthesis in adipose tissue.

#### 5.2.3. Induction of ER Stress by Mitochondrial Dysfunction and Hepatic Insulin Resistance

 Mitochondrial dysfunction induces ER stress, and this, in turn, causes hepatic insulin resistance [86]. In human liver cell lines, inhibition of mitochondrial function by oligomycin disturbs insulin signaling. In contrast, hepatic gluconeogenesis is abnormally increased. The levels of ER stress markers were elevated in cells containing functionally inactivated mitochondria. However, this rise was reversed by decreasing the level of cytosolic-free Ca^2+^. Importantly, mitochondrial dysfunction elevated the level of cytosolic-free Ca^2+^, which in turn promoted an increase in the concentrations of the ER Ca^2+^ channels IP_3_Rs and the ryanodine receptor-2 (RyR-2). Elevated levels of these channels induced Ca^2+^ depletion within the lumen of the ER. Disturbances in Ca^2+^ homoeostasis in the ER are also known to trigger the ER stress response, leading to activation of p38 mitogen-activated protein kinase (MAPK), as well as increasing phosphoenolpyruvate carboxykinase (PEPCK) expression [87, 88]. Abnormal activation of JNK by mitochondrial dysfunction also increased PEPCK expression by affecting insulin signaling and forkhead box protein O1 (FOXO1) activity [86]. Together, the results suggest that mitochondrial dysfunction induces ER stress in a Ca^2+^-dependent manner, leading to disturbance of insulin signaling and an abnormal rise in gluconeogenesis within hepatocytes.

#### 5.2.4. Induction of ER Stress by Mitochondrial Dysfunction and Local ATP Depletion

A number of events may contribute to the linking of mitochondrial dysfunction and ER stress. For example, local ATP pools in the mitochondria and the adjacent ER may be essential to supply the energy required by SERCA to import Ca^2+^ into the lumen of the ER. In agreement with this idea, inhibition of OXPHOS was shown to cause a prolonged delay in uptake of Ca^2+^ into the lumen of the ER; in addition, Ca^2+^ levels within the ER fell [89]. Inhibition of OXPHOS caused rapid local ATP depletion in mitochondria and the ER, although global cytosolic ATP levels decreased at a much later time. These results suggest that local ATP depletion in the region in which SERCA is active may reduce the uptake of Ca^2+^ into the lumen of the ER. This would cause Ca^2+^ depletion within the ER, which may trigger the ER stress response. Whether this mechanism is operative in pancreatic *β*-cells and/or insulin-responsive tissues remains to be determined.

## 6. Conclusions

**Figure 3:**
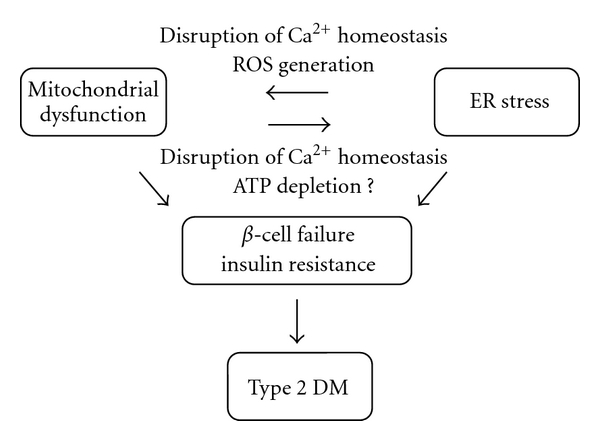
Bidirectional communication between dysfunctional mitochondria and the ER under stress contributes to the development of type 2 DM.

We have provided a brief overview of the interaction between mitochondrial dysfunction and ER stress. In particular, we examined the role played by such interaction in the pathogenesis of type 2 DM. Mitochondrial dysfunction and ER stress are essential for *β*-cell dysfunction and peripheral insulin resistance. To date, substantial progress has been made in understanding structural and functional communications between mitochondria and the ER. We now know that ER stress can induce mitochondrial dysfunction. Thus, such stress plays a central role in apoptosis signaling via Ca^2+^- and/or ROS-dependent mechanisms. Together with recent findings linking mitochondrial dysfunction and ER stress, it appears that bidirectional communication exists between these two organelles (Figure 3). Characterization of interactions between mitochondria and the ER is a dynamic and growing area of interest; future research will carefully dissect such processes. Hopefully, the studies will help us to gain a better understanding of the pathogenesis underlying type 2 DM. Therapeutic approach aimed at restoring mitochondria function will prevent or treat insulin resistance and type 2 DM through suppression of ER stress.
